# ﻿A new species of *Chryxus* Champion, with taxonomic notes on other species of the genus (Hemiptera, Heteroptera, Reduviidae, Chryxinae)

**DOI:** 10.3897/zookeys.1104.79411

**Published:** 2022-06-14

**Authors:** Hélcio R. Gil-Santana, John M. Leavengood Jr., Jean-Michel Bérenger, David dos Santos Martins, Jader Oliveira

**Affiliations:** 1 Laboratório de Diptera, Instituto Oswaldo Cruz, Av. Brasil, 4365, 21040-360, Rio de Janeiro, RJ, Brazil Laboratório de Diptera, Instituto Oswaldo Cruz Rio de Janeiro Brazil; 2 United States Department of Agriculture, APHIS, PPQ, 9325 Blay Plaza Blvd, Suite 206, Tampa Fl 33619, USA United States Department of Agriculture Tampa United States of America; 3 IRD, AP-HM, SSA, Vitrome, IHU Méditerranée Infection, Aix-Marseille Université, Marseille, France Aix-Marseille Université Marseille France; 4 Laboratoire d’Entomologie du Museum National d’Histoire Naturelle, Paris, France Laboratoire d’Entomologie du Museum National d’Histoire Naturelle Paris France; 5 Instituto Capixaba de Pesquisa, Assistência Técnica e Extensão Rural, Vitória, ES, Brazil Instituto Capixaba de Pesquisa, Assistência Técnica e Extensão Rural Vitória Brazil; 6 Universidade de São Paulo, Faculdade de Saúde Pública, Laboratório de Entomologia em Saúde Pública, São Paulo, SP, Brazil Universidade de São Paulo Sao Paulo Brazil; 7 Laboratório de Parasitologia, Universidade Estadual Paulista “Julio de Mesquita Filho”, Faculdade de Ciências Farmacêuticas UNESP/FCFAR, Rodovia Araraquara Jaú, KM 1, 14801-902, Araraquara, SP, Brazil Universidade Estadual Paulista “Julio de Mesquita Filho” Araraquara Brazil

**Keywords:** Assassin bugs, female and male genitalia, Guyana, Panama

## Abstract

*Chryxusgarcetebarretti***sp. nov.** from Paraguay is described, taxonomical notes on *C.bahianus* Gil-Santana, Costa & Marques, 2007 and *C.tomentosus* Champion, 1899 are provided; the latter species is recorded from French Guiana for the first time; a redescription of the genus *Chryxus* Champion, 1899 and an updated key for the genera and species of Chryxinae are presented.

## ﻿Introduction

Chryxinae currently includes four genera and five species of rarely collected reduviids (Lent and Wygodzinsky 1944; Weirauch 2012; Gil-Santana et al. 2015). Gil-Santana et al. (2007, 2015) summarized the taxonomic history of the group and the scant data available on the biology of this subfamily. Chryxinae can be separated from other Reduviidae by their medium or small size (3–9 mm); the head wide and anteriorly strongly curved downwards; the short, stout and strongly curved labium; and the membrane of hemelytron with only one large cell (Gil-Santana et al. 2007; Weirauch 2012; Weirauch et al. 2014).

The rarity of specimens has made the study of generic limits within the subfamily difficult, posing doubts on the validity of *Wygodzinskyella* Usinger, 1952, for example (Forero 2004; Weirauch 2012).

In the present paper, *Chryxusgarcetebarretti* sp. nov. from Paraguay is described, *Chryxus* Champion, 1899 is redescribed, taxonomical notes on *C.bahianus* Gil-Santana, Costa & Marques, 2007 and *C.tomentosus* Champion, 1899 are provided, and an updated key for the genera and species of Chryxinae is presented.

## ﻿Materials and methods

Photographs of the male holotype of *Chryxusbahianus* (Figs 2–4) were kindly provided by the team of the digitization project of the Entomological Collection of MNRJ (“Projeto Informatização da Coleção Entomológica do Museu Nacional/UFRJ, SIBBR/CNPq proc. 405588/2015–1”), taken before the fire which destroyed the collection of MNRJ, including this holotype, in 2018 (Escobar 2018). Additional images of the male genitalia of the holotype (Figs 5–9), already dissected previously, were directly produced by the first author (HRG-S).

**Figure 1.**
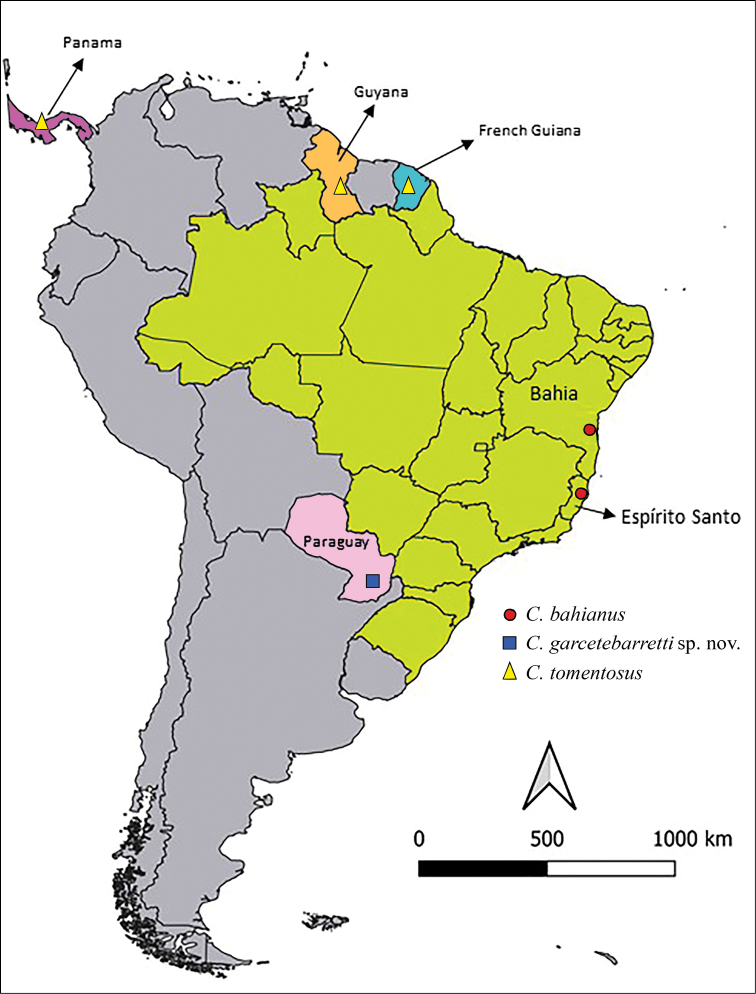
A general map of South America and a small portion of Central America (Panama, purple color) showing occurrence points of species of *Chryxus*: *C.bahianus* (red circles) in the States of Bahia and Espírito Santo of Brazil (pale green color); *C.garcetebarretti* sp. nov. (blue square) in Paraguay (pink color), and *C.tomentosus* (yellow triangles) in French Guiana (blue color), Guyana (orange) (marked randomly in the middle of the country; exactly location not recorded), and Panama (purple).

**Figures 2–9.**
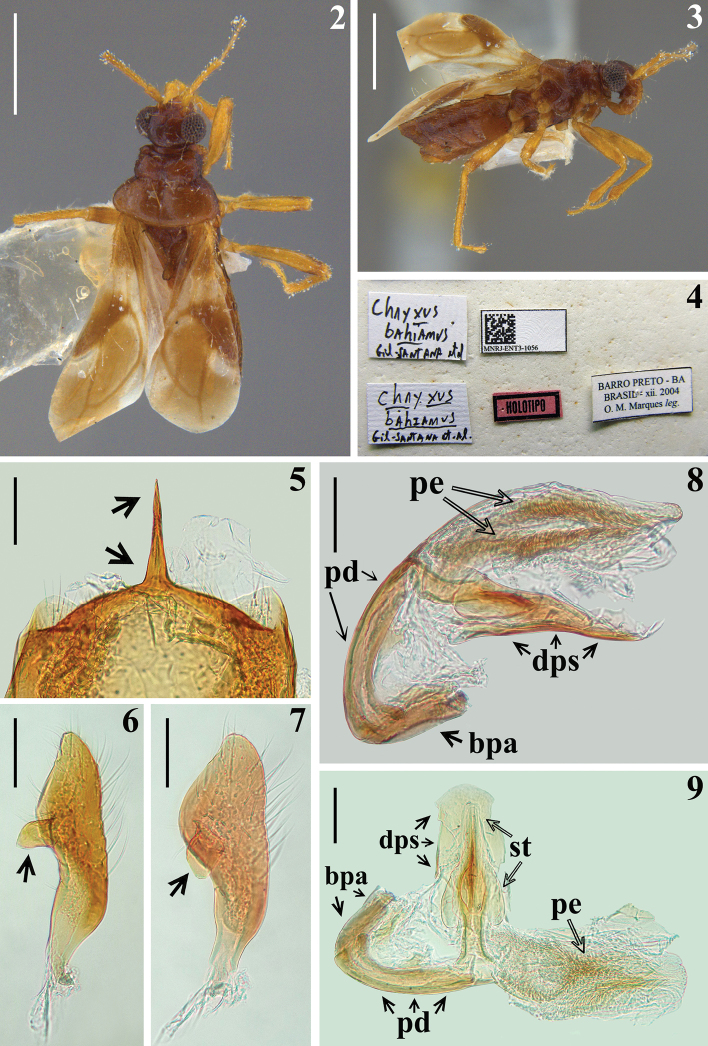
*Chryxusbahianus* Gil-Santana, Costa & Marques, 2007, male holotype, previously deposited in MNRJ**2** dorsal view **3** lateral view **4** labels **5–9** male genitalia **5** apex of pygophore and medial process of pygophore (pointed by arrows), posterior view **6, 7** paramere; laminar process pointed by an arrow **6** lateral view **7** inner surface **8, 9** phallus **8** lateral view **9** with endosoma and dorsal phallothecal sclerite set apart. Abbreviations: **bpa** basal plate arm, **dps** dorsal phallothecal sclerite, **pd** pedicel, **pe** process of endosoma, **st** struts. Scale bars: 1.0 mm (**2, 3**); 0.1 mm (**5–9**).

**Figures 10–12.**
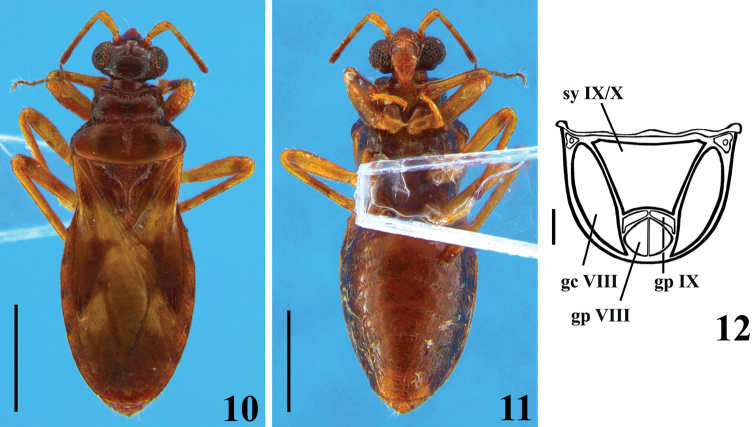
*Chryxusbahianus* Gil-Santana, Costa & Marques, 2007, female specimen from Espírito Santo State, Brazil **10** dorsal view **11** ventral view **12** external genitalia, posterior view, schematic drawing, setation omitted. Abbreviations: **sy IX/X** syntergite IX/X, **gc VIII** gonocoxa VIII, **gp VIII** gonapophysis VIII, **gp IX** gonapophysis IX. Scale bars: 1.0 mm (**10, 11**); 0.1 mm (**12**).

Photographs of a non-type female specimen of *Chryxusbahianus* (Figs 10, 11) were taken by João Paulo Sales Oliveira Correia (“Laboratório Nacional e Internacional de Referência em Taxonomia de Triatomíneos” (**LNIRTT**), Instituto Oswaldo Cruz (**IOC**), Rio de Janeiro, Brazil), with a Leica DMC 2900 camera attached to a Leica M205C stereomicroscope. Several images were stacked using the LAs software version 4.9.

Photographs of *Chryxusgarcetebarretti* sp. nov. (Figs 13–28) were taken by the second author (JMLJr.) using a Nikon Digital Sight DS-Fi2 imaging system mounted on a Nikon SMZ-18 stereomicroscope. Photograph layers were stacked using Helicon Focus 6, and composite photographs were edited using Adobe Photoshop 2020. Morphology was measured using a digital Vernier caliper.

**Figures 13–18.**
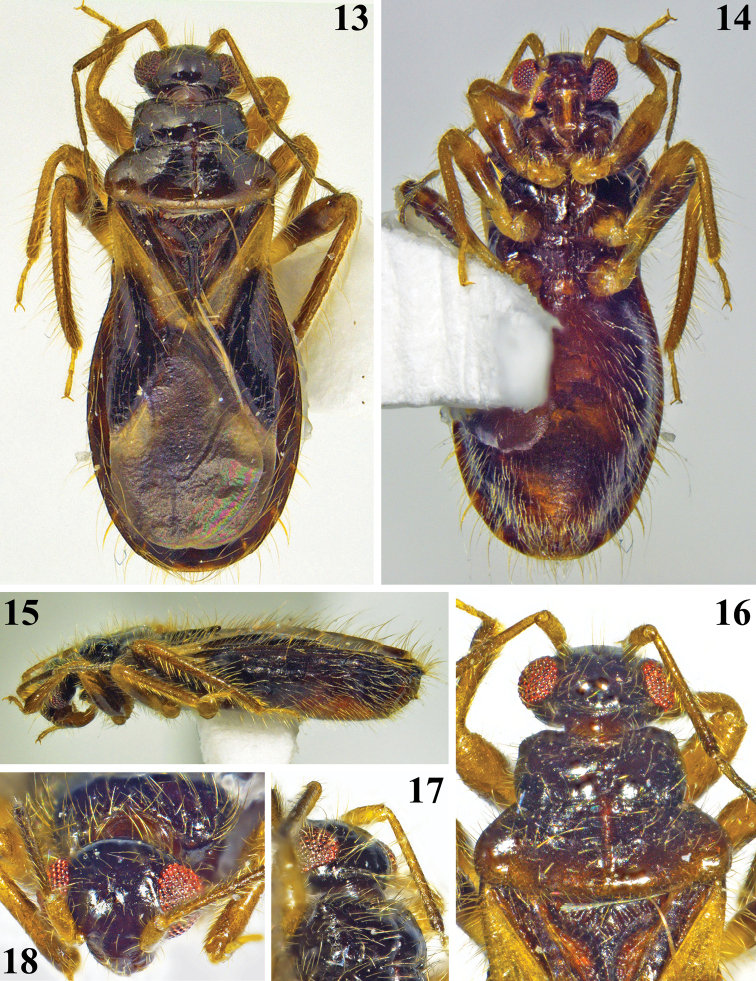
*Chryxusgarcetebarretti* sp. nov., female holotype **13** dorsal view **14** ventral view **15** lateral view **16** head, pronotum and basal portion of scutellum, dorsal view **17, 18** head and fore lobe of pronotum **17** dorsolateral view **18** frontal view.

**Figures 19–24.**
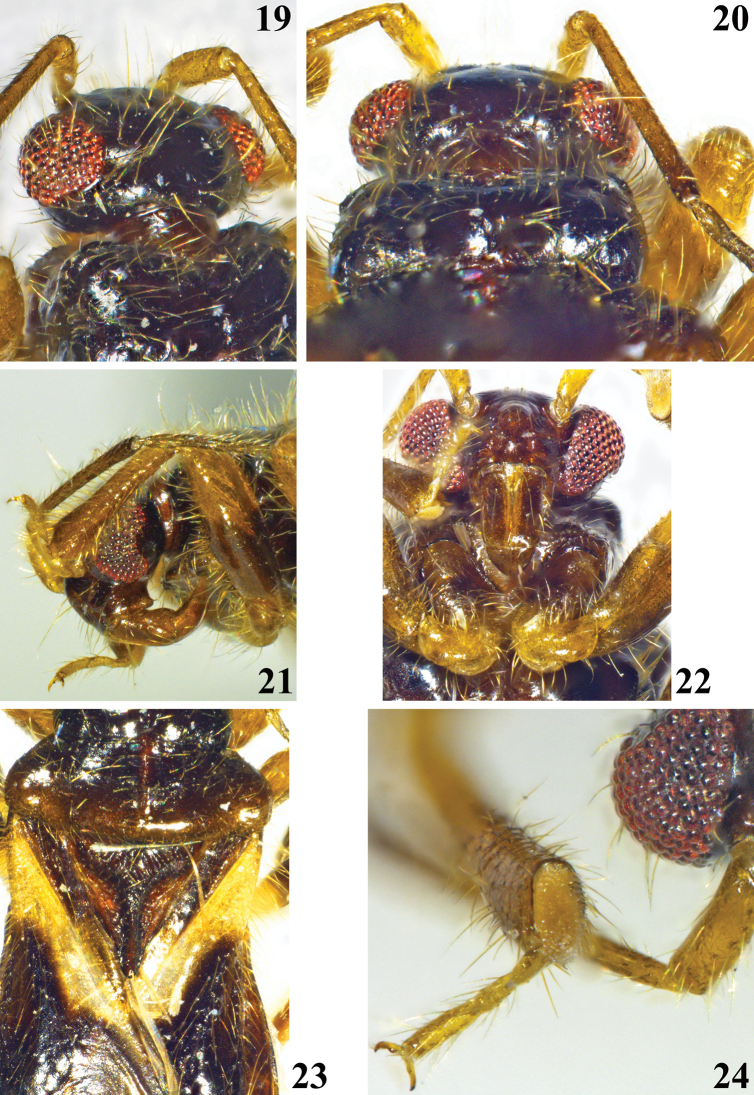
*Chryxusgarcetebarretti* sp. nov., female holotype **19, 20** head and fore lobe of pronotum **19** dorsolateral view **20** dorsoposterior view **21** head and foreleg, lateral view **22** head and fore coxae, ventral view **23** hind lobe of pronotum, scutellum and basal portion of hemelytra, dorsal view **24** apex of fore tibia, tarsus and a portion of an eye and antennal scape.

**Figures 25–28.**
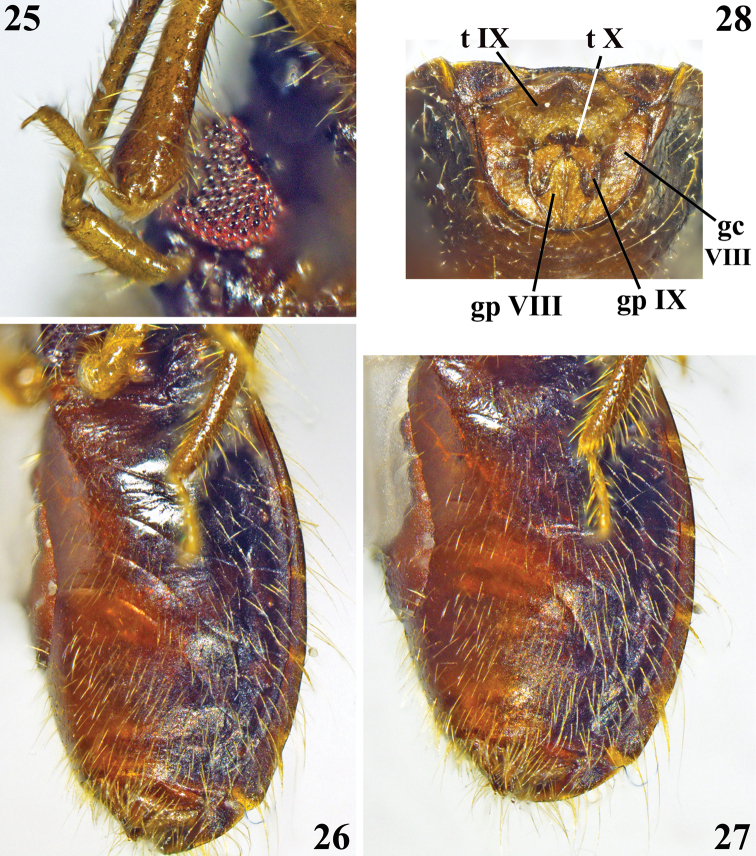
*Chryxusgarcetebarretti* sp. nov., female holotype **25** apex of fore tibia and scape, lateral view **26, 27** abdomen, ventrolateral view **28** external genitalia, posterior view. Abbreviations: **t IX** tergite IX, **t X** tergite X, **gc VIII** gonocoxa VIII, **gp VIII** gonapophysis VIII, **gp IX** gonapophysis IX.

**Figures 29–32.**
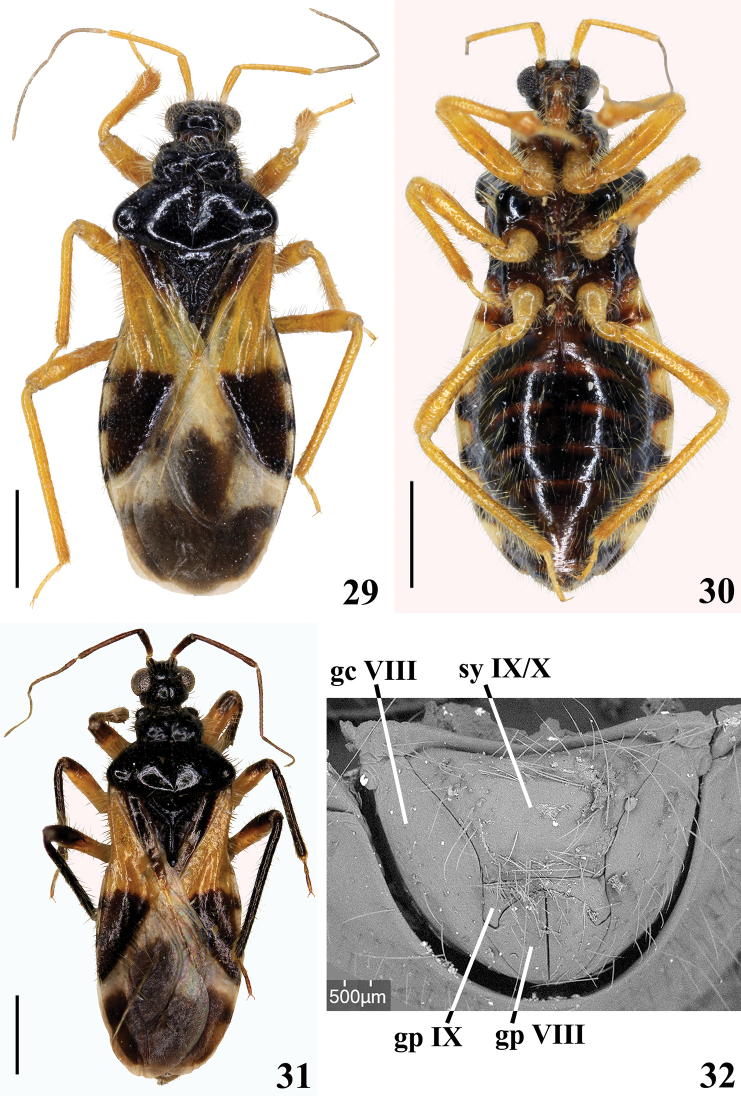
*Chryxustomentosus* Champion, 1899, females from French Guiana **29** dorsal view **30** ventral view **31** specimen from Mont Itoupé, dorsal view **32** external genitalia, posterior view, SEM image. Abbreviations: **sy IX/X** syntergite IX/X, **gc VIII** gonocoxa VIII, **gp VIII** gonapophysis VIII, **gp IX** gonapophysis IX. Scale bars: 1.0 mm (**29–31**).

Photographs of *Chryxustomentosus* (Figs 29–31) were taken by the third author (J-MB) using a Canon EOS 5D Mark II digital camera with a Laowa 25 mm ultra-macro lens. Several images were stacked using software combine ZP 1.0. S. A scanning electron microscopy image (Fig. 32) of female genitalia was obtained by the third author (J-MB) using a TM 4000 Plus Hitachi tabletop microscope.

The holotype of *Chryxusgarcetebarretti* sp. nov. (Figs 13–28) is deposited at the Florida State Collection of Arthropods (**FSCA**; Gainesville, Florida, USA). The non-type female specimen of *Chryxusbahianus* (Figs 10–12) will be deposited in the Collection of National Museum of the Federal University of Rio de Janeiro, Rio de Janeiro, Brazil (**MNRJ**).

The 17 female specimens of *Chryxustomentosus* examined are deposited in the third author’s private collection (J-MB), in France. They were collected by the Société Entomologique Antilles-Guyane (SEAG) during a study on a protected area of French Guyana. The specimens were among some important material caught using interception traps (similar to those described by Lamarre et al. 2012).

General morphological terminology mainly follows general current works on Reduviidae (e.g., Schuh and Weirauch 2020) and Chryxinae (Gil-Santana et al. 2007; Weirauch 2012).

Diagnoses of *Chryxus* and its species were not given because their characteristics are the same as described in the key presented below.

When describing label data, a slash (/) separates the lines and a double slash (//) the different labels.

## ﻿Results

### ﻿Taxonomy

**Chryxinae Champion, 1899**

#### ﻿ Chryxus

Taxon classificationAnimaliaHemipteraReduviidae

Champion, 1899

ECD62B83-349D-57C7-A219-A03FA5F04650

##### Note.

Based on two males from Panama, Champion (1899) created *Chryxus* to include a new species, *C.tomentosus*. Because *Chryxustravassosi* Lent & Wygodzinsky, 1944 was transferred to *Wygodzinskyella* by Usinger (1952), *Chryxus* was composed only of its type species and *C.bahianus* Gil-Santana, Costa & Marques, 2007 (Gil-Santana et al. 2007).

##### Redescription.

Total length 3.6–5.2 mm. Integument generally shiny and covered by numerous long and thin setae; membranes of hemelytra glabrous. ***Head*** wider than long, strongly curved anteriorly; interocular distance in dorsal view about twice the width of an eye; transverse sulcus shallow; a short anterior sulcus arising from middle of transverse sulcus, even shallower; eyes setose, coarsely faceted, widely separated from each other, globose, subhemispherical in dorsal view; clypeus moderately elevated; antennifers small, close to eyes; first two antennal segments stout; scape slightly curved, thicker (except its thinner base) and shorter than other antennal segments; remaining segments progressively thinner, generally covered by long, thin, numerous setae; on scape sparser and shorter. Labium short, stout, very curved; first two visible segments subcylindrical, subequal in length; the last segment shorter, tapering. Gena ventrally projecting in a short process. Neck well separated from head, relatively thin and short. ***Thorax*.** Pronotum: anterior collar narrow, clearly marked, lateral angles slightly or largely prominent; fore lobe subrectangular, hind lobe trapezoidal, both separated by a well-defined transverse sulcus; fore lobe shorter and narrower than hind lobe, convexly raised at disc; a median sulcus running from approximately distal portion or distal margin of fore lobe to about distal two-thirds of hind lobe; humeral angles rounded. Scutellum: basal portion with oblique ridges or wrinkled on central portion; lateral margins elevated, running towards distal process; distal process elongated, variably thickened, and obliquely elevated or not elevated at its apex. Supracoxal lobes of propleura somewhat prominent, those of meso- and metapleura progressively less or not prominent. Legs: fore coxae close to each other, separated by a distance shorter than or subequal to width of fore coxa; middle and hind coxae separated by a distance subequal to or larger than the width of respective coxae. Femora variably thickened; fore tibiae thickened towards apex, with a pad at apex; middle and hind tibiae cylindrical, straight or somewhat curved; tarsi three-segmented. Hemelytra ending short or slightly surpassing posterior margin of abdomen; membrane of hemelytron with only one central cell. ***Abdomen*** oval; connexivum moderately narrow. Sternite II finely canaliculated in both sides of middle of posterior margin.

##### Distribution.

Brazil, French Guiana (new record), Guyana, Panama, Paraguay (new record).

#### ﻿ Chryxus bahianus

Taxon classificationAnimaliaHemipteraReduviidae

Gil-Santana, Costa & Marques, 2007

FFBDFDAB-F1D4-58C8-AEE9-714DB0C7C598

Figs 1 , 2–9 , 10–12

##### Note.

*Chryxusbahianus* was described based on a single male from the State of Bahia, northeastern Brazil. Unfortunately, the holotype of *C.bahianus* was destroyed on the 2^nd^ of September 2018, during the fire which destroyed most of the zoological collections, including the entire Heteroptera collection of the MNRJ (Escobar 2018). However, images taken before the fire (Figs 2–4) in addition to the drawings presented in the original publication (Gil-Santana et al. 2007), are useful in providing a better knowledge of the holotype. Additionaly, a female (Figs 10–12) from the State of Espírito Santo, a neighbouring state of Bahia, was included to this study.

##### Type material examined.

*Chryxusbahianus*, male holotype, **Brazil**: Bahia: [handwritten labels]: *Chryxus* / *bahianus* / Gil-Santana et al // *Chryxus* / *bahianus* / Gil-Santana et. [sic] al. // [printed labels]: QR CODE / MNRJ-ENT3-1056 // BARRO PRETO - BA / BRASIL - xii. 2004 / O. M. Marques *leg.* [printed red label bordered with black lines]: HOLOTIPO [= holotype] (previously deposited in MNRJ, now destroyed).

##### Additional non-type material.

*Chryxusbahianus*, female, **Brazil**: Espírito Santo: Linhares, Reserva Natural Vale, 19°06'S, 39°45'W, 17.iii.1989, J. S. dos Santos leg., Gil-Santana det. (MNRJ).

##### Morphological remarks.

**Holotype male.** Measurements (mm): total length to tip of hemelytra: 3.6; pronotum length: 0.7; hind lobe maximum width: 1.1; abdomen maximum width: 1.2. ***Coloration*** (Figs 2, 3): ***head*** blackish; brownish on clypeus and adjacent portions; second and third visible labial segments pale brownish and dark yellowish, respectively. Scape and pedicel pale brownish; flagellomeres darkened. Neck reddish brown. ***Thorax*** blackish brown; fore coxae pale brownish; supracoxal lobes and approximately distal two thirds of middle and hind coxae dark yellowish; trochanters pale; remaining portions of legs pale brownish; femora with faint dark narrow rings on subbasal and subapical positions. Hemelytra: clavus dark brownish; corium whitish, with a subbasal dark spot and dark at approximately apical half; membrane pale brownish with two whitish markings, a basolateral spot just after apex of corium and a whitish stripe adjacent to inner margin, contiguous with whitish portion of corium, going from basal portion, shortly invading discal cell, narrowing at midportion, and enlarged at inferodistal portion; veins darkened. ***Abdomen*.** Connexivum with approximately distal third of segments III–VI darkened. Sternites reddish brown, darkened on lateral portions. ***Vestiture*** formed by golden long setae, sparse on head and thorax and somewhat more numerous on sternites (Figs 2, 3). Longer curved setae on clypeus and adjacent portion; lateral angles of pronotal collar with a single conspicuous, long, somewhat curved seta inserted in a small elevation. ***Structure*** (Figs 2, 3). Integument shiny, except dull hemelytra. Pronotum: integument generally smooth; lateral angles slightly prominent; a series of canaliculae behind anterior collar, larger at median portion; median sulcus running from just before transverse furrow to about posterior third of pronotum, larger at basal portion, where it is canaliculated, narrowing towards distal portion; transverse furrow enlarged, formed by canaliculae. Scutellum obliquely elevated and enlarged at its apex. Femora slightly thickened, fore femora a little more thickened than others; hind tibiae straight; hemelytra slightly surpassing posterior margin of abdomen. Connexival segments II–V with posterolateral acute prominences, which are progressively smaller towards distal segments. Sternite II with a shallow keel, on basal portion.

**Male genitalia** (Figs 5–9). Pygophore covered by numerous setae on exposed portion, in ventral view suboval to subsquare in shape, in lateral view dorsal margin almost straight and ventral margin rounded; medial process of pygophore thin, long, straight, spiniform in anterior and posterior views (Fig. 5), and moderately curved and more thickened in lateral view; parameres symmetrical, generally covered with moderately curved, thin, short to elongate setae (except glabrous basal (inserted) portion), enlarged at approximately middle third, on its inner face medially with a subquadrate laminar process with curved distal margin (Figs 6, 7). Phallus (Figs 8, 9): articulatory apparatus with short basal plate arms (bpa); pedicel (pd) elongated, curved in lateral view. Dorsal phallothecal sclerite (dps) faintly sclerotized, subrectangular; struts (st) fused to each other, quite enlarged at middle portion, narrowed towards distal third. Process of endosoma (pe) formed by a paired subparallel series of faintly sclerotized thickenings.

**Female.** Measurements (mm): total length to tip of abdomen: 3.7, to tip of hemelytra: 3.5; head length (excluding neck): 0.5; length of anteocular portion: 0.15; length of postocular portion: 0.05; width across eyes: 0.7; interocular distance (synthlipsis): 0.4, width of eye: 0.15; length of eye: 0.3; lengths of antennal segments: I: 0.2; II: 0.6; III: 0.4; IV: 0.3; lengths of labial segments: first visible: 0.17; second visible: 0.15; third visible: 0.17. Thorax: pronotum: fore lobe length (at midline): 0.3, (sublaterally, where it is maximum): 0.32; maximum width: 0.7; hind lobe: length: 0.4; maximum width: 1.1; scutellum, total length: 0.55; width at base: 0.6; length of hemelytra: 2.4. Fore legs: length of femur: 0.7; length of tibia: 0.7; length of spongy fossa: 0.15; length of tarsus (claws excluded): 0.35; middle legs, length of femur: 0.7; length of tibia: 0.8; length of tarsus (claws excluded): 0.3; hind legs: length of femur: 1.0; length of tibia: 1.2; tarsus absent. Abdomen, length: 1.9; maximum width: 1.3. Generally similar to male (Figs 10, 11). Setae generally less numerous and shorter. ***Head***: second and third visible labial segments brownish and pale brownish, respectively. ***Thorax***: median portion of pronotum somewhat paler; supracoxal lobes pale brownish; pleural and sternal integument generally darker, blackish; coxae dark brownish; subbasal dark ring on hind femora indistinct; fore femora slightly more thickened. Hemelytra not attaining posterior margin of abdomen (Fig. 10); veins generally darkened, except whitish inner vein of corium meeting upper portion of discal cell and respective vein enclosing pale portion of discal cell (Fig. 10). ***Abdomen***: intersegmental sutures between sternites very curved at median portion (Fig. 11). Sternite VII quite larger than preceding segments, somewhat more than twice longer at midline than sternite VI (Fig. 11). **Female genitalia.** Posterior view (Fig. 12): light brownish, gonapophysis IX somewhat paler. Syntergite IX/X large, horizontal, as inverted subtrapezoidal; gonocoxa VIII elongate, moderately curved; gonapophysis VIII subrounded; gonapophysis IX arciform.

##### Distribution

**(Fig. 1).** Brazil, states of Bahia and Espírito Santo.

#### ﻿ Chryxus garcetebarretti sp. nov.

Taxon classificationAnimaliaHemipteraReduviidae

C62C6154-8FBD-55F5-B253-EF80E69B22D2

http://zoobank.org/AF7D5827-7DA7-415E-8DC6-B2A7E1ECB4C7

Figs 1 , 13–18 , 19–24 , 25–28

##### Type material examined.

*Chryxusgarcetebarretti* sp. nov., female holotype: **Paraguay**: Misiones Dept.: San Ignacio, vic. Hotel Rural, 26°52.508'S, 56°59.355'W, 1.479 m.a.s.l., 5–8.xii.2019, Eger, Tyson & Leavengood leg. (FSCA).

##### Description.

**Holotype female.** Measurements: total length to tip of abdomen: 4.23; to tip of hemelytra: 4.04; head (excluding neck) length: 0.33; length of anteocular portion: 0.06; length of postocular portion: 0.07; width across eyes: 0.87; interocular distance (synthlipsis): 0.52; width of eye: 0.19; length of eye: 0.30; lengths of antennal segments: I: 0.36; II: 0.76; III: 0.61; IV: 0.67; lengths of labial segments: first visible: 0.29; second visible: 0.23; third visible: 0.09. Thorax: pronotum: fore lobe, length (at midline): 0.33, (sublaterally, where it is maximum): 0.39; maximum width: 0.93; hind lobe: length: 0.54; maximum width: 1.39; scutellum, total length: 0.72; width at base: 0.83; length of hemelytra: 2.88. Fore legs: length of femur: 0.98; length of tibia: 0.77; length of spongy fossa: 0.17; length of tarsus (claws excluded): 0.29; middle legs, length of femur: 1.08; length of tibia: 0.95; length of tarsus (claws excluded): 0.34; hind legs: length of femur: 1.22; length of tibia: 1.47; length of tarsus (claws excluded): 0.36. Abdomen, length: 2.34; maximum width: 1.74. ***Coloration*** (Figs 13–27): ***head*** blackish; labium brownish; scape and pedicel pale brownish, apical portion of pedicel darkened; flagellomeres darkened, basal portion of basiflagellomere paler. ***Thorax*** blackish; posterior margin of pronotum slightly paler; sclerite below basoposterior margins of scutellum reddish brown; meso- and metasterna blackish brown; coxae brown and pale on basal and distal halves respectively; trochanters pale orange to pale yellowish; legs brownish, femora pale at basal portion and largely dark to blackish at median portion; tarsi pale yellowish. Hemelytra: corium mostly blackish, basal third yellowish and whitish on anterior and posterior halves, respectively; membrane dark brownish, veins concolorous; two faint pale rounded spots just around discal cell, one basolateral just after apex of corium and another inferomedial, adjacent to inner margin. ***Abdomen*.** Connexivum dark brownish with narrow pale distal yellowish bands, which include the respective intersegmental suture; pale band between segments III and IV extending on basal portion of the latter too. Sternites blackish at lateral portion and reddish brown at median portion. ***Vestiture*.** Conspicous lateral clusters of setae at each connexival intersegmental suture, ventrally; the most dense of which between segments VI and VII (Figs 14, 26, 27). ***Structure*** (Figs 13–27). Pronotum: integument generally smooth; lateral angles slightly prominent; median sulcus narrow, margins tortuous, running from transverse furrow to near posterior margin; transverse furrow narrow. Process of scutellum with a narrow sulcus between elevated margins; apex not elevated and slightly thickened. Hind tibiae slightly curved at distal third. Connexivum with a continuous uniform margin. Intersegmental sutures between sternites very curved at median portion. Sternite VII quite larger than preceding segments, somewhat more than four times at midline than sternite VI. ***Female genitalia*.** Posterior view (Fig. 28): pale brownish with scattered darker portions. Tergite IX large, horizontal; tergite X small, surrounded by tergite IX, except posterior margin; gonocoxa VIII elongate, moderately curved; gonapophysis VIII pointed laterally at median portion; gonapophysis IX claviform.

##### Distribution

**(Fig. 1).** Paraguay, department of Misiones.

##### Etymology.

The new species is named in honor of Dr. Bolívar Rafael Garcete-Barrett (Curator of Entomology of the “Museo Nacional de Historia Natural del Paraguay”, San Lorenzo, Paraguay) for his great contribution to Entomology and specially for his indispensable help which resulted in the collection of the holotype of *C.garcetebarretti* sp. nov.

##### Comments.

The inclusion of *C.garcetebarretti* sp. nov. in *Chryxus* is in accordance with the characteristics assigned to this genus (Champion 1899; Gil-Santana et al. 2007), whereas the diagnostic characteristics recorded (see key below) seem to justify considering it as a species different from its congeners. Besides the general characteristics stated in the key, in regard to *C.bahianus*, the species to which *C.garcetebarretti* sp. nov. seems closer, the coloration of the hemelytra are different between them too. Corium mostly blackish, with basal third yellowish and whitish on anterior and posterior halves, respectively in *C.garcetebarretti* sp. nov. and whitish, with a subbasal dark spot, approximately apical half dark below the whitish area, giving the impression of a transverse pale band in *C.bahianus*. In *C.garcetebarretti* sp. nov., pale markings on membrane faint, that adjacent to the inner margin, just below discal cell, not including a portion of the latter, small and rounded, while in *C.bahianus*, pale markings of membrane more marked, whitish; that adjacent to inner margin, larger, forming a whitish stripe, contiguous with the whitish portion of the corium, going from the basal portion, shortly invading the discal cell, narrowing at midportion and enlarged at the inferodistal portion. Yet, the features of the female genitalia, as seen in posterior view (Fig. 28), are also distinctive in relation to the other species (Figs 12, 32), including *C.bahianus*, whereas the female genitalia of the latter species seems more similar to that of *C.tomentosus*.

#### ﻿ Chryxus tomentosus

Taxon classificationAnimaliaHemipteraReduviidae

Champion, 1899

531DCB34-51C8-552E-82A0-9DE9A6D086C5

Figs 1 , 29–32

##### Note.

Besides the two male syntypes from Panama (Champion 1899), only two other specimens (sex not mentioned) of *C.tomentosus* were recorded in the literature: one as being collected in Guyana (Usinger 1952) and the other in Panama (Lucas et al. 2016).

##### Non-type material examined.

**French Guiana**. Itoupé, 400 m.a.s.l., window trap n°1, 1 female, 31.iii.2010; Saül, window trap, 6 females, 10.xii.2010, 20.xii.2010, 07.iii.2011, 07.iii.2011, 22.iii.2011, 22.iii.2011, SLAM bas (Sea, Land and Air Malaise trap) (SLAM Trap-Standard BugDorm Store), 4 females, 21.iii.2012, 03.vii.2012, 31.x.2012, 27.xi.2012; Saül Belvédère, window trap, 6 females, 09.ix.2010, 17.ix.2010, 06.x.2010, 5.xi.2010, 20.xii.2010, 24.i.2011, SEAG leg. (J–MB).

##### Morphological remarks.

Measurements (mm): total length to tip of hemelytra: 4.75 to 5.2; Pronotum length: 1.25; hind lobe maximum width: 1.75; abdomen maximum width: 2.0. ***Coloration*** (Figs 29, 30): ***head*** blackish; apical half of first, second and third visible labial segments pale brownish. Scape and pedicel pale orange to yellowish; flagellomeres darkened, except paler basal portion of basiflagellomere. ***Thorax*** blackish; legs pale orange to orange yellowish. Hemelytra: corium yellowish with somewhat less than distal half blackish; clavus blackish and pale yellowish on approximately basal and distal halves, respectively; membrane pale whitish with a large blackish spot occupying almost entirely discal cell, except basal portion of discal cell, and another blackish spot in the distal region, which may be partially contiguous to the spot of discal cell. ***Abdomen*.** Connexivum pale yellow to whitish with distal markings which are larger on the ventral portion of each segment. Sternites generally blackish with some brownish stripes on segments IV–VII. All specimens show the same coloration as described above, except one specimen from Mont Itoupé with antennae, distal portion of femora, tibiae and clavus entirely black (Fig. 31). ***Structure*** (Figs 29–31). Pronotum. Fore lobe: lateral angles largely prominent; shallow faintly defined oblique furrows present along its surface; median sulcus formed by a series of foveae, which may present separately or partially fused along the sulcus and are more or less progressively smaller towards distal portion; median sulcus running from just before transverse sulcus (which is interrupted by the proximal fovea), to somewhat far from posterior margin of pronotum. Transverse furrow narrow. Scutellum with its apex elevated and somewhat thickened. Hind tibiae straight. Connexivum with a continuous uniform margin. ***Female genitalia*.** Posterior view (Fig. 32): dark blackish, gonapophysis IX paler. Syntergite IX/X large, horizontal, as inverted subtrapezoidal; gonocoxa VIII elongate, moderately enlarged at median portion; gonapophysis VIII pointed laterally at median portion; gonapophysis IX subclaviform.

##### Distribution

**(Fig. 1).** French Guiana (new record), Guyana, and Panama.

## ﻿Discussion

The Chryxinae has been considered as being rarely collected reduviids, with only one to about half a dozen specimens known of all species so far (Lent and Wygodzinsky 1944; Weirauch 2012; Gil-Santana et al. 2007, 2015). However, in our study, 17 females of *C.tomentosus*, a species from which only four specimens were previously reported (Champion 1899; Usinger 1952; Lucas et al. 2016), were assembled. They were collected in French Guiana using the windowpane trap similar to that described by Lamarre et al. (2012). It is noteworthy that only females have been collected. Only further collecting with other methods in the same area will help to clarify if the absence of males was caused by the collecting method or other factors. On the other hand, the known apparently limited distributions of species of *Chryxus* (Fig. 1), and also of other Chryxinae, may eventually reveal themselves to be much larger with future study as well as future descriptions of new species if more efficient methods of collecting them are discovered or developed in the future. It would allow a better knowledge of the group as a whole and possibly to solve taxonomic doubts about the validity and limits of their genera such as *Wygodzinskyella* (Forero 2004; Weirauch 2012).

### ﻿Key to genera and species of Chryxinae, modified from Gil-Santana et al. (2007, 2015) and Weirauch (2012)

**Table:** 

1	Total length 8.0–9.0 mm; veins on corium indistinct; connexivum with uniform clear coloration	***Wygodzinskyellatravassosi* (Lent & Wygodzinsky, 1944)**
–	Total length 3.1–5.3 mm; veins on corium distinct, at least basally; connexivum with clear and dark alternate colors	**2**
2	Head with process on frons	***Petasolentiagoellnerae* Weirauch, 2012**
–	Head without process on frons	**3**
3	Head with ocelli and an acute process on its ventral surface; corium of hemelytra with a small costal cell	***Lentiacorcovadensis* Wygodzinsky, 1946**
–	Head without ocelli or an acute process on its ventral surface; corium of hemelytra without a small costal cell	***Chryxus* Champion, 1899**…**4**
4	Fore lobe of pronotum with shallow oblique furrows and anterolateral angles largely prominent (Figs 29, 31). Connexivum pale with distal dark markings (Figs 29, 30)	***Chryxustomentosus* Champion, 1899**
–	Fore lobe of pronotum with integument generally smooth, without lateral furrows and anterolateral angles slightly prominent. Connexivum reddish brown or dark brownish with darkened or pale markings, respectively	**5**
5	Transverse and median sulci of pronotum uniformly narrow (Figs 13, 16, 23). Femora largely darkened at median portion (Figs 14, 21). Apex of scutellum not elevated (Fig. 15). Hind tibiae slightly curved at distal third (Figs 13, 14). Connexivum dark brownish with narrow pale distal yellowish bands, which include the respective intersegmental suture, margin continously uniform (Figs 13, 14, 26–27)	***Chryxusgarcetebarretti* sp. nov.**
–	Transverse and basal half of median sulci of pronotum enlarged (Fig. 2, 10). Femora with subbasal and subapical darkened rings (Figs 2, 3, 10). Apex of scutellum obliquely elevated (Fig. 2). Hind tibiae straight (Fig. 3, 10, 11). Connexival segments III–VI darkened at their distal thirds (Figs 3, 10); margins of segments II–V proeminent posterolaterally (Fig. 3)	***Chryxusbahianus* Gil-Santana, Costa & Marques, 2007**

## Supplementary Material

XML Treatment for Chryxus

XML Treatment for Chryxus bahianus

XML Treatment for Chryxus garcetebarretti

XML Treatment for Chryxus tomentosus
